# Probiotics and vitamin C for the prevention of respiratory tract infections in children attending preschool: a randomised controlled pilot study

**DOI:** 10.1038/ejcn.2014.174

**Published:** 2014-09-10

**Authors:** I Garaiova, J Muchová, Z Nagyová, D Wang, J V Li, Z Országhová, D R Michael, S F Plummer, Z Ďuračková

**Affiliations:** 1 Research and Development Department, Cultech Ltd, Port Talbot, UK; 2 Institute of Medical Chemistry, Biochemistry and Clinical Biochemistry, Faculty of Medicine, Comenius University, Bratislava, Slovakia; 3 Juvenalia Paediatric Centre, Dunajská Streda, Slovakia; 4 Department of Clinical Sciences, Liverpool School of Tropical Medicine, Pembroke Place, Liverpool, UK; 5 Divison of Computational and Systems Medicine, Department of Surgery, Imperial College, London, UK; 6 Cancer, Faculty of Medicine and Centre for Digestive and Gut Health, Institute of Global Health Innovation, Imperial College, London, UK

## Abstract

**Background:**

This pilot study investigates the efficacy of a probiotic consortium (Lab4) in combination with vitamin C on the prevention of respiratory tract infections in children attending preschool facilities.

**Subjects/methods:**

In a double-blind, randomised, placebo-controlled pilot study with children aged 3–6 years, 57 received 1.25 × 10^10^ colony-forming units of *Lactobacillus acidophilus* CUL21 (NCIMB 30156), *Lactobacillus acidophilus* CUL60 (NCIMB 30157), *Bifidobacterium bifidum* CUL20 (NCIMB 30153) and *Bifidobacterium animalis subsp. lactis* CUL34 (NCIMB 30172) plus 50 mg vitamin C or a placebo daily for 6 months.

**Results:**

Significant reductions in the incidence rate of upper respiratory tract infection (URTI; 33%, *P*=0.002), the number of days with URTI symptoms (mean difference: −21.0, 95% confidence interval (CI):−35.9, −6.0, *P*=0.006) and the incidence rate of absence from preschool (30%, *P*=0.007) were observed in the active group compared with the placebo. The number of days of use of antibiotics, painkillers, cough medicine or nasal sprays was lower in the active group and reached significance for use of cough medicine (mean difference: −6.6, 95% CI: −12.9, −0.3, *P*=0.040). No significant differences were observed in the incidence rate ratio or duration of lower respiratory tract infection or in the levels of plasma cytokines, salivary immunoglobulin A or urinary metabolites.

**Conclusions:**

Supplementation with a probiotic/vitamin C combination may be beneficial in the prevention and management of URTIs.

## Introduction

Respiratory tract infection (RTI) in children presents a considerable health-care burden involving not only the cost of direct medical care but also that incurred due to parental absence from work.^1^ Children attending preschool are three times more likely to suffer an infection than those staying at home due to high transmission rates within such facilities.^2^ RTIs can occur in the upper or lower respiratory tract affecting the sinuses, throat, airways or lungs. Antibiotics are administered for bacterial infections, but their inadvertent use for viral infections is ineffective and can contribute to antibiotic overuse and antibiotic resistance.^3^ Alternative strategies for the prevention of RTI in children attending preschool are needed.^4^

Evidence suggests that supplementation with probiotics may prevent upper respiratory tract infections (URTIs).^5^ Probiotics are defined as ‘live microorganisms that when administered in adequate amounts confer a health benefit on the host'.^6^ Some studies using probiotics alone for children attending preschool facilities have shown significant reductions in incidence and/or duration of URTIs^7, 8, 9, 10, 11^ while others observed little or no effect.^12,13^ Meta-analysis of the results from supplementation with vitamin C also suggests a beneficial effect on the duration of common cold symptoms in children.^14^

Administration of *Lactobacillus plantarum* NCIMB 8826 or *Lactobacillus reuteri* F275 prevented severe RTI in healthy mice by reducing pro-inflammatory cytokine expression at the respiratory mucosa,^15^ and an ‘anti-inflammatory priming' of immunity has been observed in peripheral blood mononuclear cells extracted from healthy humans supplemented with the Lab4 probiotic consortium.^16^ Vitamin C has also been shown to hinder the pro-inflammatory cytokine response in monocytes and lymphocytes extracted from healthy adults,^17^ but to date, no studies have examined the effect of a probiotic and vitamin C combination on RTI in preschool children. The objective of this study was to investigate the impact of a probiotic consortium in combination with vitamin C on both the incidence and duration of RTIs and assess any associated immunological and metabolic changes in children.

## Materials and methods

### Participants

Sixty-nine healthy children (3–6 years) attending preschool in Slovakia were recruited into the study. Written informed consent was obtained from parents or legal guardians prior to participation in the study. Children were excluded if they were taking medication or immunostimulatory products or any form of probiotic prior to or at the time of enrolment or if sensitive to xylitol/sorbitol. None of the children received the flu vaccine during the study period.

### Study design and intervention

A double-blinded, randomised, placebo-controlled study was conducted. The protocol was approved by the Ethical Committee of the Department of Health Care and Human Pharmacy, Trnava, Slovakia (16/09/2010) and registered with Current Controlled Trials (ISRCTN28722693). During the 6-month intervention study period, children received daily either one chewable tablet containing the Lab4 probiotic consortium comprising two strains of *Lactobacillus acidophilus* CUL21 (NCIMB 30156) and CUL60 (NCIMB 30157), *Bifidobacterium bifidum* CUL20 (NCIMB 30153) and *Bifidobacterium animalis subsp. lactis* CUL34 (NCIMB 30172) at a total of 1.25 × 10^10^ colony-forming units (*Lactobacillus* sp. 1 × 10^10^ and *Bifidobacterium* sp. 0.25 × 10^10^) and 50 mg vitamin C on a base of xylitol or an identical looking placebo tablet containing xylitol (Cultech Ltd, Port Talbot, UK). Compliance was assessed by monitoring the amount of tablets returned.

### Data collection

The children were examined by a paediatric physician and background information, including the history of allergy, was recorded. Body weight and height were measured using a digital weighing and measuring station with automatic body mass index calculation (kg/m^2^, SECA 764, SECA Deutschland, Hamburg, Germany). Twelve-hour overnight fasting venous blood samples were collected into EDTA-vacutainer tubes, and the plasma was extracted by centrifugation (1200 *g*, 10 min) before storage at −80 °C. Urine samples were collected from the first morning urination and stored at −80 °C prior to ^1^H nuclear magnetic resonance (NMR) analysis. Before saliva collection, children rinsed out their mouths with cold water, and saliva was collected in plastic tubes and stored at −20 °C. Parents/guardians completed weekly symptom diaries monitoring temperature, runny nose, sore throat, cough, chest wheezes, earache, diarrhoea, vomiting, stomach aches, absence from preschool, prescriptions of antibiotics, physician visits, hospitalisation and any medication taken during the intervention period. At the start and end of the study, they completed a dietician-designed diet habit and physical activity pattern questionnaire consisting of 3 questions on meal and drink patterns, 12 questions on intake of various foods and 3 questions relating to physical activities. The questionnaires were assessed using a scoring system, and summary scores at baseline and upon completion of the study were compared.

During the intervention period, children were examined by a paediatric physician at prescheduled 2-, 4- and 6-month appointments or during unscheduled visits resulting from URTI, LRTI or any other illness. At 6 months, blood, urine and saliva were collected. Not all children provided a saliva or blood sample on the collection days thereby reducing the active and placebo group size.

### Measurement of plasma cytokine levels

Quantification of interferon-γ (IFN-γ), interleukin (IL)-10, IL-12p70, IL-13, IL-1β, IL-2, IL-4, IL-5, IL-8 and tumour necrosis factor-α (TNF-α) was conducted using a MULTI-SPOT electrochemiluminescence array (MSD technology) in accordance with the manufacturer's protocol (CBS, Cardiff University, Cardiff, UK).

### Measurement of total salivary immunoglobulin A (IgA)

Saliva samples were centrifuged at 800 *g* for 15 min, and IgA levels were assayed by enzyme-linked immunosorbant assay (E80-102, Bethyl Laboratories, Montgomery, AL, USA).

### ^1^H NMR spectroscopic analysis of urinary samples

Urine samples were thawed at room temperature, vortexed for 10 s and centrifuged at 10 000 *g* for 10 min, and the supernatant was transferred into a 96-well plate and using a robotic system (Bruker, Rheinstetten, Germany). In all, 540 μl of urinary sample was mixed with 60 μl of 1.5 M potassium phosphate buffer in D_2_O, pH=7.4, 0.1% 3-(trimethylsilyl)-[2,2,3,3-^2^H_4_] propionic acid sodium salt (TSP) and 2 mM sodium azide and transferred into an NMR tube. ^1^H NMR spectra were acquired using a Bruker 600 MHz spectrometer (BrukerGermany) at the operating ^1^H frequency of 600.13 MHz at a temperature of 300 K. A standard NMR pulse sequence (recycle delay-90°-*t*_1_-90°-*t*_m_-90°-acquisition) provided a standard one-dimensional ^1^H NMR spectral data (*t*_1_ was 3 ms and *t*_m_ (mixing time) was 10 ms). The water peak suppression was achieved using selective irradiation during a recycle delay of 4 s and *t*_m_. A 90-degree pulse was adjusted to 10 μs. A total of 32 scans were collected into 64 k data points with a spectral width of 20 p.p.m.

### Randomisation

Eligible subjects were allocated in a 1:1 ratio to the two arms of the study according to a computer-generated random sequence using block randomisation with a block-size of four. The randomisation was performed by the study statistician, who had no contact with the participants. Participants were enrolled and assigned sequentially to interventions by the paediatric physician. The allocation sequence was not available to any member of the research team until databases had been completed and locked.

### End point measures

The primary end point measures were the incidence and duration of URTI and lower respiratory tract infection (LRTI), absence from preschool and number of visits to the paediatric centre due to RTI. URTI symptoms included sneezing, sore throat, cough, runny and blocked nose. Each distinct episode was the time period (in days) covering the continuous display of symptoms, separated from another episode by a minimum of 24 h. LRTI was confirmed by a paediatric physician, and LRTI duration was the number of days between the physician confirmed onset and absence of symptoms. Secondary end points were changes in plasma cytokines, salivary IgA and urine metabolites. Parent-reported measures included diarrhoea (⩾3 loose stools in a 24-h period), vomiting and stomach aches.

### Data management and analysis

As a pilot study there was no a formal sample size calculation. For the primary end point analysis, incidence rate ratio (number of episodes divided by the number of days in the study) and mean difference in the duration of URTIs and LRTIs, absence from preschool and number of visits to paediatric centre for RTIs during the intervention period with 95% confidence intervals (CIs) were calculated using a generalised linear model (GLM) that included treatment as a single predictor. The secondary immunological end points were analysed similarly. For the GLM analysis of a continuous end point such as duration of URTI and LRTI, normal distribution and identity link functions were used. For the GLM analysis of recurrence of events (such as the number of episodes of URTI symptoms), Poisson distribution and log link functions were used. *Post-hoc* covariate-adjusted analyses within the GLM framework were performed on the primary end points with treatment as study variable and centre, age, gender and body mass index as covariates. Subgroup analysis was performed by gender. Continuous variables were summarised using the number of observations, mean (s.d.), whereas categorical variables were summarised by the number and percentage of events.

Analysis of study end points was performed in per protocol analysis (PP) and a modified intention-to-treat (ITT) population excluding the participants who withdrew shortly after randomisation. Dietary habit and physical exercise questionnaire data analysis was performed using the GLM model, with treatment as study variable and baseline measurement as covariate. Data analyses were performed using SAS version 9.2 (SAS Institute Inc., Cary, NC, USA).

### ^1^H NMR data analysis

Automatic phasing, baseline correction and reference to TSP signal at δ^1^H 0.00 were performed on the ^1^H NMR urinary spectra. The processed NMR spectral data (δ^1^H 0.25–10) were imported to MATLAB (R2012a, 7.14.0.739, MathWorks) and digitised into 20 k data points with a resolution of 0.0005 p.p.m. using a MATLAB script developed within the Section of Computational and Systems Medicine. The water peak region (δ^1^H 4.7–4.9) was removed owing to its disordered peak shape caused by water suppression. In addition, variations of the intensity of urea signal (δ^1^H 5.4–6.1) can be caused by chemical exchange with the solvent; hence the region was also removed. Probabilistic quotient normalisation was performed on the remaining spectral data in order to account for dilution of complex biological mixtures. Principal component analysis and orthogonal partial least squares discriminant analysis were carried out on the unit variance-scaled data in THE SIMCA (P+13.0) and MATLAB software. Orthogonal partial least squares regression analyses were performed to correlate metabolic data with cytokine measurement.

## Results

### Enrolment and baseline characteristics

**Figure 1:**
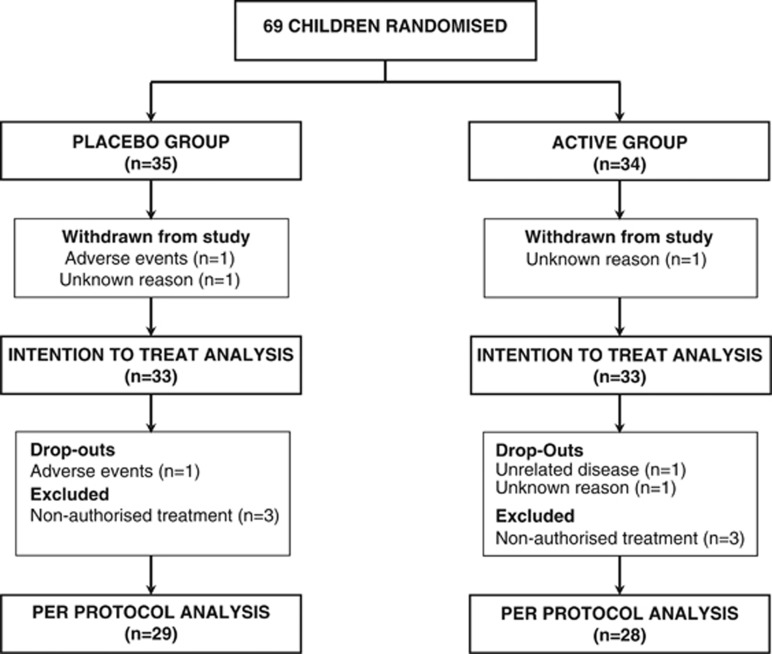
Participant's flow chart.

Sixty-nine children were enrolled between October 2010 and March 2011; 35 into the placebo group and 34 into active. Three children did not provide any records and withdrew from the study. Nine children were excluded from the PP analysis; six due to non-authorised treatment usage and three due to non-completion of the follow-up period (Figure 1). As can be seen in Table 1, the baseline data for both groups appears comparable. The cohort consists of 16 girls and 17 boys per group with the mean age of 5 years. In all, 12% of children in the placebo group and 6% in active group had atopic diseases.

### Primary end points

There was a significant reduction in the duration of total URTI symptoms (days) in the active group compared with the placebo (PP analysis; mean difference: −21.0, 95% CI:−35.9, −6.0, *P*=0.006, ITT analysis; mean difference: −17.9, 95% CI:−31.6, −4.2, *P*=0.011; Table 2). The incidence rate of URTI symptoms in the active group was 0.0196 compared with 0.0293 in the placebo group (33% reduction, 95% CI in reduction: 14%, −48%, *P*=0.002, PP analysis; Table 3). A similar result was seen when an ITT analysis was performed.

In all, 35.7% of children in the active group experienced ⩾3 different URTI symptoms per URTI episode compared with 51.7% of children in the placebo group (risk difference: 16%, 95% CI: −9.4%, 41.4%, *P*=0.217, PP analysis). There were no significant differences in the duration or incidence rate ratio of LRTIs (Table 4).

The incidence rate of absence from preschool in the active group was 0.0198; 30% lower than in the placebo group (0.0282, *P*=0.007, PP analysis), and there was a trend towards significance for the number of days absent from preschool and the number of paediatric physician visits due to URTI symptoms (*P*=0.070 and *P*=0.082, respectively, Table 2, PP analysis) in the active group compared with placebo group. No significant differences in the number of days absent from preschool or physician visits due to LRTI were observed, and there was no hospitalisation due to RTIs during the course of the study. The results from *post-hoc* covariate-adjusted analysis are similar to those from unadjusted analysis (Supplementary Tables S3–S5).

### Secondary end points

There were no significant differences in plasma cytokine levels between groups, but there were significant reductions in IL-12p70, IL-13, IL-1β, IL-2, IL-4, IL-5, IL-8, IL-10 and TNF-α between baseline and 6 months (data not shown). IFN-γ levels and IL-2/IL-5 were significantly reduced in the active group after 6 months (IFN-γ *P*=0.035, mean difference: −3.1; 95% CI: −6.0, −0.2 and IL-2/IL-5; *P*=0.023; mean difference: −0.2; 95% CI: −0.5, −0.04, PP analysis) but not for the placebo group. There were no significant differences in the IgA levels between the groups (*P*=0.438; mean difference: 31.2; 95% CI: −50.3, 112.8, PP analysis).

**Figure 2:**
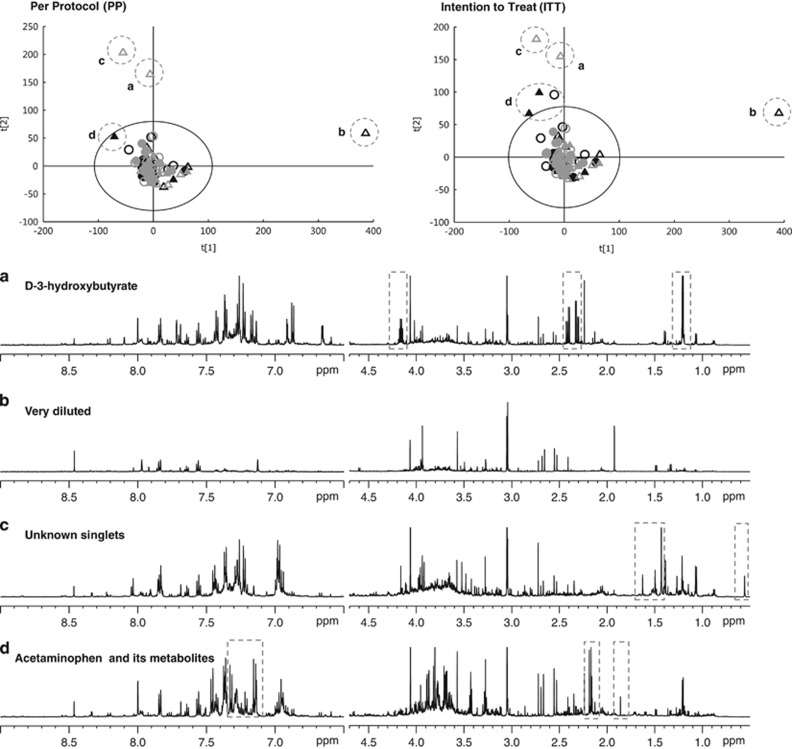
The upper plots illustrated PCA scores plots derived from ^1^H NMR spectra of urinary samples collected from children at baseline (black) and after 6 months intervention (grey) in per protocol (PP) analyses and intention to treat (ITT), respectively. Open triangles for girls placebo group; closed triangles for girls active group; open circles for boys placebo group; closed circles boys active group. Six strong outliers are observed, and their NMR spectra are shown in panels **a**–**d**. Outlier (**a**) contains a high concentration of D-3-hydroxybutyrate. Outlier (**b**) is due to its extreme dilution of the sample. Outlier (**c**) shows a set of unknown singlets at 0.54, 0.43 and 1.62 p.p.m. Group (**d**) exhibit signals of acetaminophen and its metabolites.

The principal component analysis score plots of the urine metabolic profiles, including outliers, are presented in Figure 2, but no significant changes were observed with or without outliers. Orthogonal partial least squares regression analyses were used to statistically correlate metabolic profiles with measured cytokine levels, but no significant correlation was obtained. A set of representative ^1^H NMR spectra are shown in Supplementary Figure S1.

### Additional parameters, use of antibiotics and other medications

There were no significant differences in either the incidence rate or the number of days with earache, chest wheeze, temperature >37 °C, stomach ache, vomiting or diarrhoea between the groups. The observed number of days of antibiotic, painkiller, cough medicine, nasal spray/drop or antihistamine usage was lower in the active group compared with the placebo and was significant for cough medicine usage (mean difference: −6.6, 95% CI: −12.9, −0.3, *P*=0.040, PP analysis). Seven of the 29 children (24.1%) received oral antibiotics in the placebo group compared with 4 of the 28 children (14.3%) in the active group (*P*=0.356, PP analysis). There was no hospitalisation due to RTIs during the course of the study.

### Subgroup analysis on the basis of gender

A significant reduction in number of days with URTI symptoms was observed in boys (*P*=0.024; mean difference: −25.6; 95% CI: −47.7, −3.4, PP analysis) compared with the girl groups (*P*=0.084; mean difference: −17.6; 95% CI: −37.5, 2.4, PP analysis) (Supplementary Table S1). No significant differences in the duration or incidence of LRTI symptoms were observed between genders (Supplementary Table S2).

Significant decreases or trends toward significance were observed in IL-12p70 (*P*=0.060, 95% CI: −0.6, 0.0), IL-12p70/IL-10 (*P*=0.025, 95% CI: −0.3, −0.02), IL-12p70/IL-5 (*P*=0.032, 95% CI: −2.0, −0.1) and IL-2/IL-10 (*P*=0.025, 95% CI: −0.3, −0.02) for boys in the active group compared with the placebo (PP analysis). No significant changes in the immune parameters were observed in the girls group.

### Compliance, dietary assessment, adverse events

Mean compliance to the product for the whole group was 90.8%. No significant differences in dietary habits and physical activity patterns were observed over the study period (*P*=0.625). Two children receiving placebo reported abdominal pain or vomiting and withdrew from the study.

## Discussion

Children supplemented with a combination of the Lab4 probiotic consortium and vitamin C for 6 months attending preschool facilities showed a reduced incidence and duration of URTI symptoms. Studies with probiotics alone have shown variable results for URTIs, with some reporting significant reductions in incidence and duration^7, 8, 9, 10, 11^ while others observed little or no effect.^12,13^ Positive effects on URTI have been reported in response to *Lactobacillus casei* DN-114 001, *Lactobacillus rhamnosus* GG and for *Lactobacillus acidophilus* NCFM alone or in combination with *Bifidobacterium animalis sp lactis* Bi-07 at doses ranging from 10^9^ to 10^10^ colony-forming units per day^7, 8, 9, 10, 11^ while limited evidence exists suggesting that supplementation with low dose vitamin C (<0.2 g per day) may reduce common cold duration (discussed by Hemila *et al.*^14^). A probiotic consortium combined with multi-vitamins and minerals in adults showed a 13.6% reduction in the incidence of combined URTI and LRTI symptoms.^18^ There was no impact on LRTI or gastrointestinal symptoms in this study although benefits have been reported elsewhere.^7,8,19,20^

Children receiving the Lab4/vitamin C combination had fewer days of absence from preschool and unscheduled visits to the paediatric physician, suggesting that combined supplementation may reduce the severity of infections. Reductions in preschool absence have been reported with probiotics^8, 9, 10^ and with a vitamin C (150 mg per day), echinacea and propolis combination.^20^ Fewer children receiving the supplement were treated with oral antibiotics similar to that seen in other probiotic studies,^8,10^ and there was a significant reduction in the number of days that cough medicine was used which highlights the potential socio-economic benefits associated with the combination supplement.

Metabonomics is a new approach providing a systematic analysis of the chemical products or metabolites in biological samples, such as urine, blood and faeces, and is considered as a very sensitive measure of an organism's phenotype.^21^ In this study, no changes in the urinary metabolite profiles of healthy preschool children were observed between the placebo and active groups. The major metabolites identified in ^1^H NMR spectral data agreed with those observed in healthy children.^22^

Both probiotics and vitamin C are known to modulate the immune system,^23,24^ and the combination probably mediates a response through immune-modulation although the absence of any significant changes in cytokines levels between the active and placebo groups in our study may suggest the existence of an alternate mechanism. Although there were no significant differences in salivary IgA levels between the active group and placebo, Cáceres *et al.*^13^ observed increased faecal IgA levels in *Lactobacillus rhamnosus* HN001-supplemented children but was unable to associate this with any improvements in URTI symptoms. The trend towards a reduction in IL-2/IL-5 and IFN-γ levels may suggest a shift towards an anti-inflammatory state in the supplemented group, and this is consistent with the abilities of both the Lab4 consortium and vitamin C to induce an anti-inflammatory response in immune cells extracted from the blood of healthy subjects.^16,17^

Subgroup analysis of our population on the basis of gender revealed reduced URTI symptoms in boys that corresponded with reduced plasma levels of IL-12p70, IL-12p70/IL-10, IL-2/IL-10 and IL-12/IL-5, indicating a more anti-inflammatory basal immune status. Positive effects of both probiotics and vitamin C on RTIs have been seen in adolescent and adult male subjects,^25, 26, 27^ and it is known that X-chromosome-specific immune-modulatory genes mediate male-specific immunological responses during prepubescence.^28^

As this was a pilot study with a small number of participants, it was not powered to detect all statistically significant differences, and an unwillingness to provide blood or saliva reduced the samples size for the secondary end point analysis. Also, the impact of the intervention at the time of an infection was not evaluated in this study.

## Conclusions

Supplementation with a probiotic consortium comprising *L. acidophilus* CUL21 and CUL60, *Bifidobacterium bifidum* CUL20, *Bifidobacterium animalis subsp. lactis* CUL34 and vitamin C may provide a strategy to reduce the incidence of URTIs in 3–6 year old children attending preschool facilities. These results are encouraging and need to be confirmed in a larger study population.

## Figures and Tables

**Table 1:** Baseline demographics

	*Placebo* (N*=33*)	*Active* (N*=33*)	*All* (N*=66*)
*Gender (n)*			
* Girls*	16	16	32
* Boys*	17	17	34
			
*Age (years)*^a^	5.0 (0.7)	4.9 (0.8)	5.0 (0.9)
* Girls*	4.9 (0.8)	4.8 (0.9)	4.8 (0.8)
* Boys*	5.2 (0.6)	5.0 (0.8)	5.1 (0.7)
			
*BMI (kg/m*^*2*^)^a^	15.6 (2.5)	15.5 (2.2)	15.5 (2.3)
* Girls*	15.3 (2.0)	15.8 (2.4)	15.5 (2.2)
* Boys*	15.9 (2.9)	15.2 (2.0)	15.5 (2.5)
			
*Centre, n (%)*			
* *Centre 1	21 (63.6)	21 (63.6)	42 (63.6)
Centre 2	6 (18.2)	6 (18.2)	12 (18.2)
Centre 3	6 (18.2)	6 (18.2)	12 (18.2)
			
Eczema, *n* (%)	5 (15.2)	2 (6.1)	7 (10.6)
Atopic disease,^b^ *n* (%)	4 (12.1)	2 (6.1)	6 (9.1)
Food allergy, *n* (%)	3 (9.1)	0 (0)	3 (4.5)
Coeliac disease, *n* (%)	0 (0)	1 (3.0)	1 (1.5)

Abbreviation: BMI, Body Mass Index.

a Data are presented as mean (s.d.), intention to treat analysis.

b Allergic rhinitis, atopic eczema or asthma.

**Table 2:** Duration of URTI symptoms, absence and paediatric physician visits

	*PP analysis*		*ITT analysis*	
	*Placebo* (N*=29*)	*Active* (N*=28*)	*Placebo* (N*=33*)	*Active* (N*=33*)
*URTI symptoms*				
Mean (s.d.), days	43.1 (35.4)	22.1 (21.0)	41.5 (34.5)	23.5 (21.8)
Mean difference (95% CI)	−21.0 (−35.9,−6.0)		−17.9 (−31.6,−4.2)	
*P-*value	0.006		0.011	
				
*Individual URTI symptoms*				
Sneezing				
Mean (s.d.), days	9.6 (14.8)	2.3 (4.0)	9.0 (14.1)	3.8 (8.6)
Mean difference (95% CI)	−7.4 (−12.9, −1.8)		−5.1 (−10.7, 0.4)	
*P*-value	0.010		0.069	
				
*Cough*				
Mean (s.d.), days	23.5 (20.3)	11.9 (10.1)	22.3 (19.8)	14.3 (14.4)
Mean difference (95% CI)	−11.6 (−19.8, −3.4)		−7.9 (−16.2, 0.3)	
*P-*value	0.006		0.058	
				
*Runny nose*				
Mean (s.d.), days	21.4 (25.5)	11.5 (15.6)	19.8 (24.6)	12.3 (15.8)
Mean difference (95% CI)	−10.0 (−20.8, 0.9)		−7.4 (−17.2, 2.4)	
*P-*value	0.072		0.138	
				
*Blocked nose*				
Mean (s.d.), days	9.8 (23.8)	4.9 (7.8)	10.4 (23.0)	5.4 (8.4)
Mean difference (95% CI)	−5.0 (−14.1, 4.1)		−5.0 (−13.2, 3.2)	
*P-*value	0.285		0.230	
				
*Sore throat*				
Mean (s.d.), days	2.8 (4.2)	1.9 (2.7)	3.2 (4.3)	1.8 (2.6)
Mean difference (95% CI)	−0.9 (−2.7, 0.9)		−1.4 (−3.1, 0.3)	
*P-*value	0.332		0.010	
				
*Absence, physician visits*				
*Absence from preschool due to URTI*				
Mean (s.d.), days	14.2 (18.4)	7.5 (8.0)	13.9 (17.7)	7.7 (8.6)
Mean difference (95% CI)	−6.7 (−14.0, 0.5)		−6.1 (−12.8, 0.5)	
*P*-value	0.070		0.069	
				
*Number of physician visit due to URTI*				
Mean (s.d.)	2.9 (3.1)	1.6 (2.2)	2.8 (3.0)	1.8 (2.4)
Mean difference (95% CI)	−1.2 (−2.6, 0.2)		−0.9 (−2.2, 0.4)	
*P-*value	0.082		0.164	

Abbreviations: CI, confidence interval; ITT, intention to treat; PP, per protocol; URTI, upper respiratory tract infection.

**Table 3:** Incidence rate of URTI symptoms and absence

	*PP analysis*		*ITT analysis*	
	*Incidence rate ratio (95% CI)*	P*-value*	*Incidence rate ratio (95% CI)*	P*-value*
*URTI symptoms*	0.67 (0.52, 0.86)	0.002	0.68 (0.54, 0.86)	0.002
				
*Individual URTI symptoms*				
Sneezing	0.29 (0.19, 0.47)	<0.001	0.35 (0.23, 0.53)	<0.001
Cough	0.55 (0.40, 0.76)	<0.001	0.60 (0.45, 0.81)	0.001
Runny nose	0.64 (0.47, 0.87)	0.005	0.66 (0.50, 0.89)	0.005
Blocked nose	1.15 (0.69, 1.91)	0.600	1.01 (0.64, 1.59)	0.966
Sore throat	0.70 (0.39, 1.26)	0.235	0.63 (0.37, 1.08)	0.095
				
*Absence*				
** **Absence from preschool	0.70 (0.55, 0.91)	0.007	0.68 (0.54, 0.87)	0.002

Abbreviations: CI, confidence interval; ITT, intention to treat; PP, per protocol; URTI, upper respiratory tract infection.

**Table 4:** Duration and incidence of LRTI confirmed by paediatric physician

	*PP analysis*		*ITT analysis*	
	*Placebo* (N*=29*)	*Active* (N*=28*)	*Placebo* (N*=33*)	*Active* (N*=33*)
*Number of days*				
Mean (s.d.), days	1.0 (3.4)	0.5 (1.8)	0.9 (3.2)	0.9 (3.5)
Mean difference (95% CI)	−0.6 (−2.0, 0.8)		0.03 (−1.6, 1.6)	
*P-*value	0.425		0.970	
				
*Incidence rate ratio (95% CI)*	0.5 (0.1, 2.8)		1.0 (0.2, 4.0)	
*P-*value	0.447		0.989	

Abbreviations: CI, confidence interval; ITT, intention to treat; LRTI, lower respiratory tract infection; PP, per protocol.

## Notes

The study was sponsored by Cultech Ltd, UK. IG, DRM and SFP are employees of Cultech Ltd and had no role in the recruitment, data collection and analysis. The remaining authors declare no conflict of interest.
