# Supplementary material for: Probiotics and vitamin C for the prevention of respiratory tract infections in children attending preschool: a randomised controlled pilot study
Source: Eur J Clin Nutr. 2014 Sep 10;69(3):373–9. doi: 10.1038/ejcn.2014.174 (PMC4351422; doi:10.1038/ejcn.2014.174)
Supplement: Supplementary Figure S1 [file ejcn2014174x1.doc]

**Supplementary Figure 1:** Typical 600 MHz ^1^H NMR spectra of urine obtained from children involved in the double-blinded, randomised, placebo-controlled study at baseline (A-D) and 6 months post intervention (E-H). (A, E) girls control group; (B, F) girls active group; (C, G) boys control group; (D, H) boys active group.

**Supplementary Table 1: Duration of URTI symptoms and absence from preschool on the basis of gender**

| **Boys** | | | **Girls** | | | |  |  |
| --- | --- | --- | --- | --- | --- | --- | --- | --- |
|  | **Placebo** | **Active** | |  | **Placebo** | **Active** |  |  |
|  | (N=13) | (N=16) | |  | (N=16) | (N=12) |  |  |
| **URTI symptoms** |  |  | |  |  |  |  |  |
| Mean (SD), days | 48.3 (39.6) | 22.8 (22.7) | |  | 38.8 (32.3) | 21.3 (19.4) |  |  |
| Mean difference (95% CI)  *P* value | -25.6 (-47.7,-3.4)  0.024 | | |  | -17.6 (-37.5, 2.4)  0.084 | |  | |
|  |  |  | |  |  |  |  |  |
| *Individual URTI symptoms* |  |  | |  |  |  |  |  |
| **Sneezing** |  |  | |  |  |  |  |  |
| Mean (SD), days | 10.8 (14.6) | 1.1 (1.8) | |  | 8.6 (15.2) | 3.8 (5.5) |  |  |
| Mean difference (95% CI)  *P* value | -9.7 (-16.7, -2.8)  0.006 | | |  | -4.9 (-13.6, 3.9)  0.275 | |  |  |
| **Cough** |  |  | |  |  |  |  |  |
| Mean (SD), days | 24.4 (18.4) | 12.3 (9.7) | |  | 22.8 (22.3) | 11.3 (11.0) |  |  |
| Mean difference (95% CI)  *P* value | -12.1 (-22.1, -2.0)  0.019 | | |  | -11.5 (-24.8, 1.8)  0.089 | |  |  |
| **Runny nose** |  |  | |  |  |  |  |  |
| Mean (SD), days | 19.8 (24.0) | 13.7 (17.4) | |  | 22.8 (27.4) | 8.6 (13.1) |  |  |
| Mean difference (95% CI)  *P* value | -6.2 (-20.7, 8.4)  0.407 | | |  | -14.2 (-30.4, 2.0)  0.087 | |  |  |
| **Blocked nose** |  |  | |  |  |  |  |  |
| Mean (SD), days | 16.2 (33.8) | 3.8 (6.8) | |  | 4.7 (9.0) | 6.3 (9.0) |  |  |
| Mean difference (95% CI)  *P* value | -12.3 (-28.7, 4.0)  0.138 | | |  | 1.6 (-4.9, 8.1)  0.637 | |  |  |
| **Sore throat** |  |  | |  |  |  |  |  |
| Mean (SD), days | 3.4 (5.5) | 2.5 (3.1) | |  | 2.4 (2.9) | 1.2 (2.1) |  |  |
| Mean difference (95% CI)  *P* value | -0.9 (-3.9, 2.1)  0.568 | | |  | -1.2 (-3.1, 0.7)  0.209 | |  |  |
|  |  |  | |  |  |  |  |  |
| **Absence from preschool** | | | | | | |  |  |
| Mean (SD), days | 23.5 (25.2) | 10.3 (12.0) | |  | 15.1 (15.1) | 11.7 (10.9) |  |  |
| Mean difference (95% CI)  *P* value | -13.2 (-26.7, 0.2)  0.054 | | |  | -3.4 (-13.1, 6.4)  0.495 | |  |  |

Abbreviations: PP, Per Protocol; N, number; SD, Standard deviation; URTI, Upper Respiratory Tract Infections

**Supplementary Table 2: Duration and incidence of LRTI confirmed by paediatric physician** on the basis of gender

|  | **Boys** | |  | **Girls** | |
| --- | --- | --- | --- | --- | --- |
|  | **Placebo** | **Active** |  | **Placebo** | **Active** |
|  | (N=13) | (N=16) |  | (N=16) | (N=12) |
| **Number of days** |  |  |  |  |  |
| Mean (SD), days | 2.0 (4.9) | 0.0 (0.0) |  | 0.3 (1.0) | 1.1 (2.6) |
| Mean difference (95% CI) | -2.0 (-4.3, 0.3) | |  | 0.8 (-0.5, 2.2) | |
| *P* value | 0.093 | |  | 0.223 | |
| **Incidence Rate Ratio** (95% CI) | 0.0 (0.00, 0.00) | |  | 2.67 (0.24, 29.41) | |
| *P* value | 0.999 | |  | 0.423 | |

Abbreviations: LRTI, Lower Respiratory Tract Infections; SD, standard deviation

**Supplementary Table 3: Post-hoc covariate adjusted analysis for the duration of URTI symptoms, absence and paediatric physician visits with treatment as study variable and centre, age, gender and BMI as covariates.**

| **PP analysis** | | | **ITT analysis** | | | |  |  |
| --- | --- | --- | --- | --- | --- | --- | --- | --- |
|  | **Placebo** | **Active** | |  | **Placebo** | **Active** |  |  |
|  | (N=29) | (N=28) | |  | (N=33) | (N=33) |  |  |
| **URTI symptoms** |  |  | |  |  |  |  |  |
| Mean (SD), days | 43.1 (35.4) | 22.1 (21.0) | |  | 41.5 (34.5) | 23.5 (21.8) |  |  |
| Mean difference (95% CI)  *P* value | -21.4 (-35.0,-7.7)  0.002 | | |  | -18.8 (-31.3,-6.3)  0.003 | |  | |
|  |  |  | |  |  |  |  |  |
| *Individual URTI symptoms* |  |  | |  |  |  |  |  |
| **Sneezing** |  |  | |  |  |  |  |  |
| Mean (SD), days | 9.6 (14.8) | 2.3 (4.0) | |  | 9.0 (14.1) | 3.8 (8.6) |  |  |
| Mean difference (95% CI)  *P* value | -7.3 (-12.7, -1.8)  0.010 | | |  | -5.3 (-10.7, 0.1)  0.056 | |  |  |
| **Cough** |  |  | |  |  |  |  |  |
| Mean (SD), days | 23.5 (20.3) | 11.9 (10.1) | |  | 22.3 (19.8) | 14.3 (14.4) |  |  |
| Mean difference (95% CI)  *P* value | -11.9 (-19.5, -4.3)  0.002 | | |  | -8.8 (-16.5, -1.1)  0.026 | |  |  |
| **Runny nose** |  |  | |  |  |  |  |  |
| Mean (SD), days | 21.4 (25.5) | 11.5 (15.6) | |  | 19.8 (24.6) | 12.3 (15.8) |  |  |
| Mean difference (95% CI)  *P* value | -9.7 (-19.3, -0.1)  0.047 | | |  | -8.0 (-16.5, 0.6)  0.068 | |  |  |
| **Blocked nose** |  |  | |  |  |  |  |  |
| Mean (SD), days | 9.8 (23.8) | 4.9 (7.8) | |  | 10.4 (23.0) | 5.4 (8.4) |  |  |
| Mean difference (95% CI)  *P* value | -5.6 (-14.6, 3.4)  0.223 | | |  | -5.2 (-13.3, 2.9)  0.205 | |  |  |
| **Sore throat** |  |  | |  |  |  |  |  |
| Mean (SD), days | 2.8 (4.2) | 1.9 (2.7) | |  | 3.2 (4.3) | 1.8 (2.6) |  |  |
| Mean difference (95% CI)  *P* value | -1.1 (-2.9, 0.7)  0.237 | | |  | -1.42 (-3.1, 0.2)  0.089 | |  |  |
|  |  |  | |  |  |  |  |  |
| *Absence, physician visits* |  |  | |  |  |  |  |  |
| **Absence from preschool due to URTI** | | | | | | |  |  |
| Mean (SD), days | 14.2 (18.4) | 7.5 (8.0) | |  | 13.9 (17.7) | 7.7 (8.6) |  |  |
| Mean difference (95% CI)  *P* value | -7.1 (-14.3, 0.2)  0.055 | | |  | -6.6 (-13.1, -0.1)  0.045 | |  |  |
| **Number of physician visit due to URTI** | | | | | | |  |  |
| Mean (SD) | 2.9 (3.1) | 1.6 (2.2) | |  | 2.8 (3.0) | 1.8 (2.4) |  |  |
| Mean difference (95% CI)  *P* value | -1.1( -2.5, 0.2)  0.108 | | |  | -1.0 (-2.2, 0.3)  0.137 | |  |  |

Abbreviations: PP, Per Protocol; ITT, Intention to Treat; N, number; SD, Standard deviation; URTI, Upper Respiratory Tract Infections

**Supplementary Table 4: Post-hoc covariate adjusted analysis for the incidence rate of URTI symptoms and absence with treatment as study variable and centre, age, gender and BMI as covariates.**

|  | **PP analysis** | |  | **ITT analysis** | |
| --- | --- | --- | --- | --- | --- |
|  | **Incidence Rate Ratio (95% CI)** | ***P* value** |  | **Incidence Rate Ratio (95% CI)** | ***P* value** |
|  |  |  |  |  |  |
| **URTI symptoms** | 0.7 (0.5, 0.9) | 0.004 |  | 0.7 (0.5, 0.9) | 0.001 |
|  |  |  |  |  |  |
| *Individual URTI symptoms* |  |  |  |  |  |
| **Sneezing** | 0.3 (0.2, 0.5) | <0.001 |  | 0.3 (0.2, 0.5) | <0.001 |
| **Cough** | 0.5 (0.4, 0.7) | <0.001 |  | 0.6 (0.4, 0.8) | <0.001 |
| **Runny nose** | 0.6 (0.5, 0.9) | 0.006 |  | 0.6 (0.5, 0.8) | 0.002 |
| **Blocked nose** | 1.2 (0.7, 2.0) | 0.480 |  | 1.0 (0.6, 1.6) | 0.962 |
| **Sore throat** | 0.7 (0.4, 1.2) | 0.213 |  | 0.7 (0.4, 1.1) | 0.111 |
|  |  |  |  |  |  |
| *Absence* |  |  |  |  |  |
| **Absence from preschool** | 0.7 (0.5, 0.9) | 0.005 |  | 0.7 (0.5, 0.9) | 0.001 |

Abbreviations: PP, Per Protocol; ITT, Intention to Treat; URTI, Upper Respiratory Tract Infections

**Supplementary Table 5: Post-hoc covariate adjusted analysis for the** **duration and Incidence of LRTI confirmed by paediatric physician with treatment as study variable and centre, age, gender and BMI as covariates.**

|  | **PP analysis** | |  | **ITT analysis** | |
| --- | --- | --- | --- | --- | --- |
|  | **Placebo** | **Active** |  | **Placebo** | **Active** |
|  | (N=29) | (N=28) |  | (N=33) | (N=33) |
| **Number of days** |  |  |  |  |  |
| Mean (SD), days | 1.0 (3.4) | 0.5 (1.8) |  | 0.9 (3.2) | 0.9 (3.5) |
| Mean difference (95% CI) | -0.6 (-2.0, 0.8) | |  | 0.1 (-1.5, 1.6) | |
| *P* value | 0.388 | |  | 0.937 | |
| **Incidence Rate Ratio** (95% CI) | 0.5 (0.1, 2.8) | |  | 1.0 (0.2, 3.9) | |
| *P* value | 0.449 | |  | 0.967 | |

Abbreviations: LRTI, Lower Respiratory Tract Infections; PP, Per Protocol; ITT, Intention to Treat; SD, standard deviation
